# Perceived Emotional Threat and OCD Symptoms in a Nonclinical Sample: Examining Group Differences Between People with High vs. Low OCD symptoms

**DOI:** 10.1192/j.eurpsy.2026.11605

**Published:** 2026-07-31

**Authors:** T. Accinni, P. Cordellieri, A. Quaglieri, G. D. Kotzalidis, M. Panfili, A. Maraone, M. Pasquini, A. Buzzanca

**Affiliations:** 1 Department of Human Neurosciences; 2 Department of Psychology, Sapienza University of Rome; 3 Department of Human and Social Science, Universitas Mercatorum; 4 Department of Neuroscience, Mental Health and Sense Organs, Sapienza University of Rome, Rome, Italy

## Abstract

**Introduction:**

Research highlights the role of emotional processing in Obsessive-Compulsive Disorder (OCD). This study examines differences in perceived emotional threat among individuals with varying obsessive-compulsive (OC) symptom levels in a nonclinical sample.

**Objectives:**

To examine Perceptions of Threats from Emotion Questionnaire (PTEQ, 2006) subscale differences between groups with lower and higher OC symptoms, controlling for anxiety, responsibility, somatic awareness, thought–action fusion, education, and age.

**Methods:**

Participants (n=460) completed the PTEQ, Padua Inventory (PI, 1988), Beck Anxiety Inventory (BAI, 1988), Responsibility Attitude Scale (RAS, 2000), Self-Awareness Questionnaire (SAQ, 2014), and Thought–Action Fusion Scale (TAF, 1996). Participants were split in two groups (below/above mean), and PTEQ subscale differences were examined controlling for BAI, RAS, SAQ, TAF, education, and age.

**Results:**

A statistically significant difference was found in PTEQ subscale scores based on participants’ PI scores, controlling for covariate measures and demographic factors (Wilks’ Λ=0.700, F(8,233)=12.47, *p*<.001; see Table 1). Covariate measures and demographics also significantly affected responses across emotional subscales (Table 2). Univariate tests showed significant group differences on Sadness (F=65.29, *p*<.001), Happiness (F=31.59, *p*<.001), Anger (F=34.10, *p*<.001), Fear (F=34.75, *p*<.001), Disgust (F=34.07, *p*<.001), Guilt (F=50.02, *p*<.001), Lust (F=22.41, *p*<.001), and Strong Emotion (F=60.73, *p*<.001).

**Table 1.** Multivariate Tests for Group Differences on PTEQ Subscales Table 1. Long description.

Test	Value	F	df	p
Pillai’s Trace	0.2998	12.47	8 / 233	< .001
Wilks’ Lambda	0.700	12.47	8 / 233	< .001
Hotelling’s Trace	0.4281	12.47	8 / 233	< .001
Roy’s Largest Root	0.4281	12.47	8 / 233	< .001

**Table 2.** Multivariate Effects of Covariates Table 2. Long description.

Covariate	Wilks’ Lambda	F	df	p
BAI (Anxiety)	0.825	6.19	8 / 233	< .001
RAS (Responsibility)	0.126	26.16	8 / 233	< .001
SAQ (Somatic Awaren)	0.916	4.88	8 / 233	0.008
TAF (Thought-Action)	0.904	8.52	8 / 233	0.002
Education Level	0.761	6.57	8 / 233	0.012
Age	0.818	11.44	8 / 233	< .001

**Conclusions:**

Individuals with higher OC symptoms (>mean PI score) exhibit increased perception of emotional threat compared to those with lower symptoms (<mean PI). They reported significantly greater threat linked to sadness, happiness, anger, fear, disgust, guilt, lust, and strong emotion. Findings suggest a link between obsessionality and emotion dysregulation, underscoring the relevance of perceived emotional threat in OCD, taking into account anxiety, interoceptive awareness, responsibility beliefs, thought–action fusion, and demographic factors.

**Disclosure of Interest:**

None Declared

## Long descriptions

### Table 1. Long description

The table consists of five columns: Test, Value, F, d f, and p. It contains four rows of multivariate test results, all showing highly significant results with p less than .001 and an F value of 12.47 with degrees of freedom 8 / 233.

* Pillai’s Trace: Value 0.2998, F 12.47, d f 8 / 233, p less than .001.

* Wilks’ Lambda: Value 0.700, F 12.47, d f 8 / 233, p less than .001.

* Hotelling’s Trace: Value 0.4281, F 12.47, d f 8 / 233, p less than .001.

* Roy’s Largest Root: Value 0.4281, F 12.47, d f 8 / 233, p less than .001.

Navigate back to Table 1..

### Table 2. Long description

The table consists of five columns: Covariate, Wilks’ Lambda, F, d f (degrees of freedom), and p (significance level). All covariates share the same d f value of 8 / 233.

* B A I (Anxiety): Wilks’ Lambda 0.825, F 6.19, p less than .001.

* R A S (Responsibility): Wilks’ Lambda 0.126, F 26.16, p less than .001.

* S A Q (Somatic Awareness): Wilks’ Lambda 0.916, F 4.88, p 0.008.

* T A F (Thought-Action): Wilks’ Lambda 0.904, F 8.52, p 0.002.

* Education Level: Wilks’ Lambda 0.761, F 6.57, p 0.012.

* Age: Wilks’ Lambda 0.818, F 11.44, p less than .001.

Navigate back to Table 2..

